# Improvement of symptoms in clinically suspect arthralgia and resolution of subclinical joint inflammation: a longitudinal study in patients that did not progress to clinical arthritis

**DOI:** 10.1186/s13075-020-2102-9

**Published:** 2020-01-16

**Authors:** Robin M. ten Brinck, Debbie M. Boeters, Hanna W. van Steenbergen, Annette H. M. van der Helm-van Mil

**Affiliations:** 1 Department of Rheumatology, Leiden University Medical Centre, PO Box 9600, 2300 RC Leiden, The Netherlands; 2 Department of Rheumatology, Erasmus Medical Centre, Rotterdam, The Netherlands

**Keywords:** Rheumatoid arthritis, Imaging, Outcome measures, Clinical research

## Abstract

**Introduction:**

Arthralgia and MRI-detected subclinical inflammation can precede the development of clinically evident rheumatoid arthritis (RA). However, part of the patients presenting with clinically suspect arthralgia (CSA) do not progress to RA. In these ‘non-progressors’, we aimed to study the frequencies of spontaneous improvement of arthralgia and its relation with the course of subclinical inflammation.

**Methods:**

Between April 2012 and April 2015, 241 patients were considered at risk for RA based on the clinical presentation and included in the CSA cohort. One hundred fifty-two patients with complete data on clinical follow-up did not develop clinical arthritis, of which 98 underwent serial 1.5T MRI scans (wrist, MCP2–5, and MTP1–5 joints) at baseline and after 2 years. MRI scans were scored for synovitis, tenosynovitis, and bone marrow oedema (summed: MRI inflammation score). MRI scores were compared to scores of symptom-free persons.

**Results:**

After a 2-year follow-up, 33% of the ‘non-progressors’ had complete resolution of symptoms; 67% had no symptom resolution and were diagnosed as persistent CSA (44%), osteoarthritis (10%), and tendinomuscular complaints (13%). With symptom-free controls as a reference, patients without resolution did not have increased MRI scores at any time point. However, patients achieving resolution of symptoms had increased MRI inflammation scores at baseline (4.0 vs. 2.6, *p* = 0.037), but not after 2 years (3.0 vs. 2.6; *p* = 0.57), and during follow-up, their MRI inflammation score decreased significantly (*p* = 0.036).

**Conclusions:**

A subgroup of CSA patients that did not progress to RA had spontaneous improvement of symptoms and resolution of subclinical joint inflammation. This time relationship suggests that symptoms and inflammation were causally related in these patients. Further research is needed to identify the mechanisms underlying the resolution of inflammation.

## Introduction

Rheumatoid arthritis (RA) can be preceded by a phase of a preclinical disease with signs and symptoms, in which joint swelling cannot yet be identified through physical examination [1]. More than 90% of patients that develop RA had MRI-detected subclinical inflammation in small joints in the symptomatic phase of clinically suspect arthralgia (CSA). However, of all patients that are identified as having CSA, a large part (up to 80%) do not progress to clinically evident RA [1]. Thus far, most longitudinal studies performed in patients considered at risk for RA focussed on the progression from arthralgia to RA [1, 2], since (early) identification of individuals that will develop RA is a key point from a clinician’s perspective. However, there is also a group of patients that were considered at risk for RA but over time do not develop RA, meaning that in hindsight, they possibly have not been truly ‘pre-RA’. This subgroup of patients is unexplored, and the course and outcome of joint symptoms and subclinical inflammation in these patients are yet unknown. From a clinical perspective, knowledge of the course of these symptoms could be useful. Moreover, despite non-progression, subclinical joint inflammation could be present in (part of) these patients at first presentation and comprehension on the natural course and severity of subclinical inflammation, and its relationship with spontaneous disappearance of arthralgia increases our understanding on spontaneous resolution occurring in patients at risk phases of RA.

Longitudinal studies performed in the disease phase of early undifferentiated arthritis (UA) have shown that clinical synovitis resolved spontaneously in 10–40%, without intervention with disease-modifying antirheumatic drugs (DMARDs) [3, 4]. Based on these data, it can be hypothesized that a similar (or even larger) percentage of patients with CSA will show spontaneous resolution of joint symptoms. In addition, as arthralgia is associated with the presence of local subclinical inflammation [5], it could be hypothesized that there is a causal relation and that resolution of symptoms is connected to the improvement of subclinical inflammation presuming. Furthermore, it could be presumed that patients with persistent symptoms had more severe subclinical inflammation at presentation and during follow-up compared to patients with symptom resolution.

We aimed to increase understanding of the course of symptoms in patients that presented with CSA but did not progress to RA. Therefore, the percentage of patients with symptom resolution and with persistent symptoms during a 2-year follow-up was determined. The scores of MRI-detected inflammation, and the time relationship with the evanescence of symptoms, were studied. Finally, MRI data were compared to MRI data obtained from age-matched symptom-free persons from the general population to estimate if MRI-detected joint inflammation returned to normal values.

## Methods

### Patients

Between April 2012 and April 2015, 241 patients were included in the CSA cohort: CSA patients had no clinically evident arthritis, but recent-onset (< 1 year) arthralgia of small joints, that was clinically considered at risk for RA by the rheumatologist at first presentation at the outpatient clinic. The cohort has been described before in [6]. Routine follow-up visits were performed at 4, 12, and 24 months. If necessary (for instance, when the patient experienced more symptoms or noticed a swollen joint), patients were seen in between scheduled visits by their rheumatologist. Hence, logistics were arranged such that patients in this cohort had very easy access to rheumatologic care; should a patient develop clinically evident IA, this was identified at the first opportunity. None of the patients was treated with DMARDs (including corticosteroids) during the course of the study. At the baseline visit, IgG ACPA (EliA CCP (anti-CCP2), Phadia, Nieuwegein, the Netherlands) and IgM RF (as described previously, in-house ELISA [7]) were determined. The cut-off for ACPA positivity was > 7 U/mL, and for RF positivity, it was > 3.5 IU/mL.

**Fig. 1:**
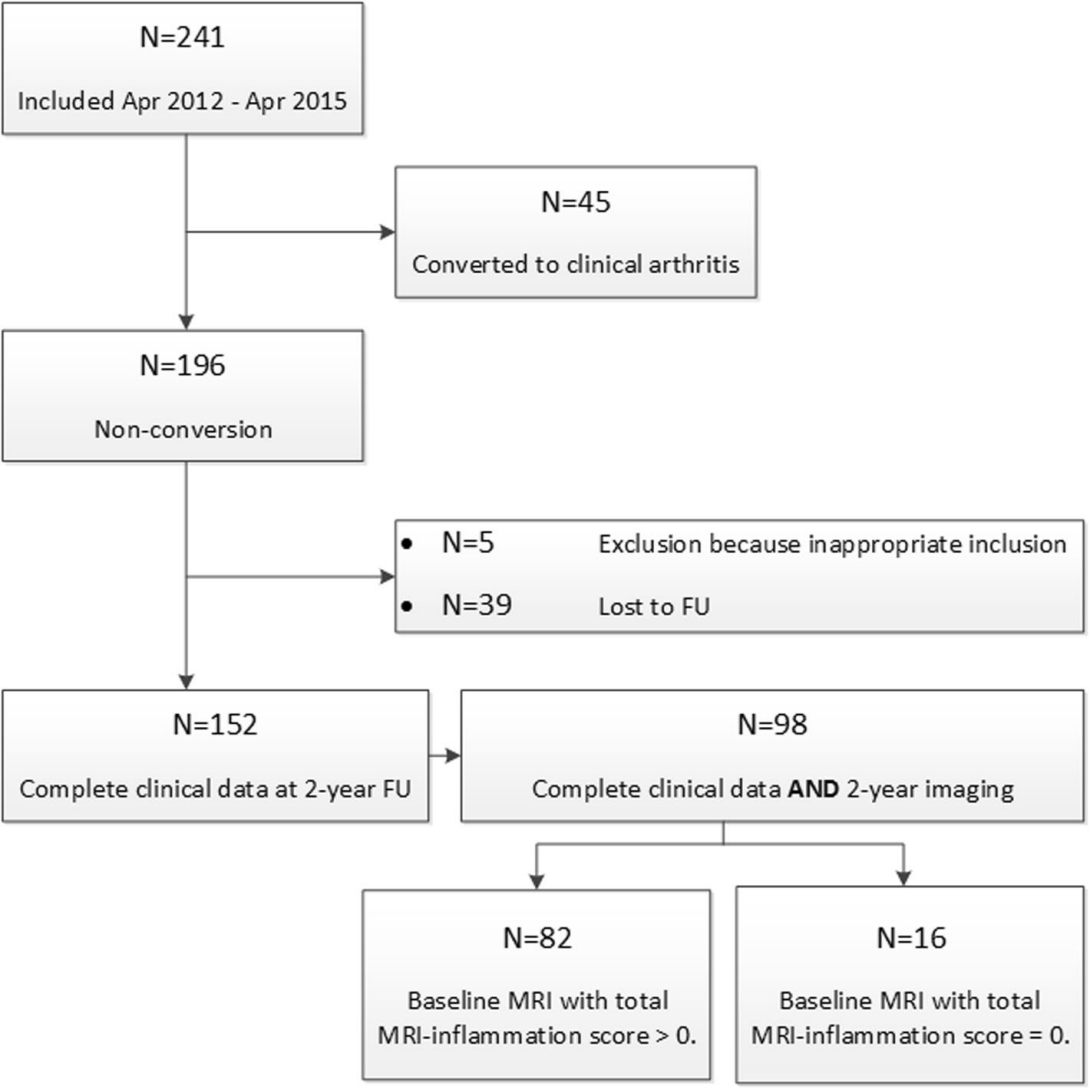
Flowchart of the different patient populations. FU, follow-up

A flowchart of inclusion is provided in Fig. 1. As this study focused on patients that did not convert to RA over time, 45 patients that were diagnosed with RA during follow-up (clinical synovitis identified at the physical examination by experienced rheumatologists, 19% out the total *n* = 241) were excluded. From the subsequent total of 196 eligible patients, 44 patients were excluded because of inappropriate inclusion (*n* = 5) or were lost to follow-up during the 2-year course of the study (*n* = 39). This resulted in complete clinical and follow-up data in 152 patients. Of these, 98 patients also had complete serial imaging data at a 2-year follow-up. Reasons for incomplete serial imaging were contra-indications for contrast-enhanced MR imaging and not willing to undergo (repeated) MR imaging. Indications of potential selection bias at the different stages of the flowchart (*n* = 241: all patients presenting with CSA, vs. *n* = 196: eligible non-converting patients, vs. *n* = 152: non-converting patients with complete follow-up data, vs. *n* = 98: non-converting patients with complete follow-up data and serial imaging) were evaluated by comparing the baseline characteristics between different patient groups.

All patients provided written informed consent. Ethics approval was provided by the local medical ethical committee.

### Assessment of symptom resolution

The main outcome was patient-reported resolution of symptoms. This was assessed at the routine follow-up visits by asking patients to answer a written question if they considered their symptoms completely resolved or not (by literally inquiring ‘are your symptoms still present’, yes or no). Patients in whom initial presenting features were resolved, but with new joint symptoms, were classified in the non-resolution group. Resolution of any related symptom (as judged by patients themselves) at the 24-month visit was used as a definition for symptom resolution.

In addition to this main outcome, pain scores on a visual analogue scale (VAS; scale 0–10) were collected to evaluate the robustness of the main outcome; the course in VAS pain was also studied. Furthermore, 68 tender joint counts (68-TJC) were studied. After 2 years without conversion to clinical arthritis, patients were mostly referred back to their GP with a clinical conclusion, unless rheumatologist and/or patients felt that longer follow-up at the rheumatology outpatient clinic was required. The clinical diagnosis after 2 years was also studied.

### Symptom-free persons

To make inferences on the presence and severity of MRI-detected subclinical inflammation as compared to the general population, MRI data from the 98 CSA patients were matched to the data of MRI-detected subclinical inflammation from symptom-free persons [8]. Matching was based on age in a 1:1 ratio, since age was previously proven to influence the severity of MRI-detected subclinical inflammation [9]. Since sex was previously demonstrated to have no effect on MRI-detected inflammation [8, 10], matching was not performed on sex. The 98 symptom-free persons had no history of inflammatory rheumatic diseases, no joint symptoms during the last month, and no evidence of synovitis at physical examination. The symptom-free persons were recruited from the general population, as described in [8].

### MRI

Unilateral MRIs of the wrist, MCP2–5, and MTP1–5 were performed at presentation with CSA (most painful or in case of equally severe symptoms the dominant side) and at 2-year follow-up (when follow-up ended) of that same side. An ONI MSK Extreme 1.5T MRI scanner (GE Healthcare, WI, USA) was used, as described previously [1] and in Additional file 1: Patients were instructed not to use NSAIDs 24 h prior to MRI, with 22 patients reporting daily use of NSAIDs at baseline. MRIs were evaluated for bone marrow oedema (BME; range 0–72), synovitis (range 0–33) [11], and tenosynovitis (range 0–54) [12]. These 3 features were summed in the total MRI inflammation score. Each MRI was scored by 2 readers, who belonged to a pool of 4 experienced readers (all had interclass correlations ≥ 0.90, see Additional file 1: Table S1). The mean scores of the 2 readers were studied. All readers were blinded to the clinical data and the order in time. MRI data were never reported to the clinicians in any phase of the study. Additional information on the scoring method is provided in Additional file 1:

### Analyses

Unpaired *t* tests were used to compare patients with symptom-free persons. For analyses over time, paired *t* tests were used. To evaluate if MRI inflammation scores changed over time, analyses using measures of MRI-detected subclinical inflammation were confined to patients with a baseline total MRI inflammation score of > 0, as a baseline score of 0 would not be able to further decrease. Eighty-two patients (84%) had a baseline MRI with a total MRI inflammation score > 0 (Fig. 1).

For consistency, total MRI inflammation scores on group level for the same 82 patients were compared to scores of age-matched symptom-free persons. Furthermore, a sub-analysis within autoantibody-positive (ACPA- and/or RF-positive; 19% of patients) CSA patients was applied. Finally, sensitivity analyses were performed on the patients meeting the EULAR definition of arthralgia suspicious for progression to RA with ≥ 3 points (*n* = 63) [13]. Statistical analyses were carried out using the Statistical Package for the Social Sciences (SPSS; version 23.0). *p* values < 0.05 were considered significant. Total MRI inflammation scores are reported as mean.

## Results

### Patient characteristics

The baseline characteristics of patients at the different stages in the flowchart (Fig. 1) did not show relevant differences, as shown in Table 1. Baseline characteristics of the patients with complete clinical follow-up and MRI data at baseline and at 2-year follow-up (*n* = 98) are demonstrated in Table 2. Patients presenting with CSA that did not progress to RA were female in 74%, had a mean age of 47 years, and a median 68-TJC of 5 joints, and 19% carried RA-related autoantibodies (RF and/or ACPA). These characteristics are comparable with previous reports on patients from the Leiden CSA cohort [1, 14], although the percentage of autoantibody-positive patients was lower in this study in non-progressors, since the presence of autoantibodies is a risk factor for progression to RA [1, 14] and autoantibodies were thus less often observed in the non-converting patients. MRI-detected inflammation was not associated with increased C-reactive protein levels (*p* = 0.38).

**Table 1:** Comparison of baseline patient characteristics between different stages of the flowchart as presented in Fig. 1

Patient characteristics	*N* = 241	*N* = 196	*N* = 152	*N* = 98
Age in years, mean (SD)	44 (13)	44 (13)	45 (13)	47 (13)
Female sex, *N* (%)	187 (78)	152 (77)	118 (78)	73 (74)
Family history of RA, *N* (%)	71 (30)	52 (27)	43 (28)	28 (29)
Symptom duration in weeks, median (IQR)	18 (10–48)	17 (9–30)	17 (9–33)	17 (9–43)
Presence of morning stiffness ≥ 60 min, *N* (%)	80 (33)	61 (35)	49 (32)	29 (30)
68-TJC, median (IQR)	6 (3–10)	6 (2–11)	6 (2–10)	5 (2–10)
VAS pain score, median (IQR)	5 (3–7)	5 (3–7)	5 (3–7)	5 (3–6)
*≥* 3 items on EULAR definition of arthralgia suspicious for progression to RA [13], *N* (%)	178 (74)	141 (72)	100 (66)	63 (64)
Increased CRP (≥ 5 mg/L), *N* (%)	53 (22)	39 (20)	29 (19)	19 (19)
Autoantibody status				
Negative for IgM-RF and ACPA, *N* (%)	184 (76)	166 (84)	125 (82)	79 (81)
ACPA- or RF-positive, *N* (%)	57 (24)	31 (16)	27 (18)	19 (19)

*ACPA* anti-citrullinated peptide antibody (positive if ≥ 7 U/mL), *CRP* C-reactive protein, *IgM-RF* immunoglobulin M rheumatoid factor (positive if ≥ 3.5 IU/mL), *IQR* interquartile range, *RA* rheumatoid arthritis, *SD* standard deviation, *TJC* tender joint count

**Table 2:** Baseline patient characteristics of the clinically suspect arthralgia patients with complete clinical follow-up (*N* = 152) and complete clinical follow-up as well as MRI data at baseline at 2-year follow-up (*N* = 98)

Patient characteristics	Complete clinical follow-up (*N* = 152)		Complete clinical follow-up and MRI data (*N* = 98)	
	Symptom resolution (*n* = 57)	No symptom resolution (*n* = 95)	Symptom resolution (*n* = 32)	No symptom resolution (*n* = 66)
Age in years, mean (SD)	44 (13)	46 (13)	46 (14)	47 (13)
Female sex, *N* (%)	40 (70)	79 (82)	20 (63)	53 (80)
Family history of RA, *N* (%)	17 (30)	26 (27)	10 (31)	18 (27)
Symptom duration in weeks*, median (IQR)	17 (9–30)	17 (9–41)	18 (15–32)	17 (9–50)
Morning stiffness ≥ 60 min, *N* (%)	22 (39)	27 (28)	10 (31)	19 (29)
68-TJC*, median (IQR)	5 (2–8)	6 (2–12)	4 (2–7)	6 (2–13)
≥ 4 tender joints, *N* (%)	33 (58)	61 (64)	18 (56)	43 (65)
Increased CRP (≥ 5 mg/L), *N* (%)	12 (21)	17 (18)	9 (28)	10 (15)
Autoantibody status				
Negative for IgM-RF and ACPA, *N* (%)	43 (75)	71 (75)	25 (78)	54 (82)
ACPA- or RF-positive, *N* (%)	9 (16)	18 (19)	7 (22)	12 (18)
ACPA-positive, *N* (%)	5 (9)	6 (6)	3 (9)	4 (6)

*ACPA* anti-citrullinated peptide antibody (positive if ≥ 7 U/mL), *CRP* C-reactive protein, *IgM-RF* immunoglobulin M rheumatoid factor (positive if ≥ 3.5 IU/mL), *IQR* interquartile range, *RA* rheumatoid arthritis, *SD* standard deviation, *TJC* tender joint count, *VAS* visual analogue scale

*Missing data were as follows: symptom duration in weeks (*n* = 4) and 68-TJC (*n* = 1)

### Resolution of symptoms over time

**Fig. 2:**
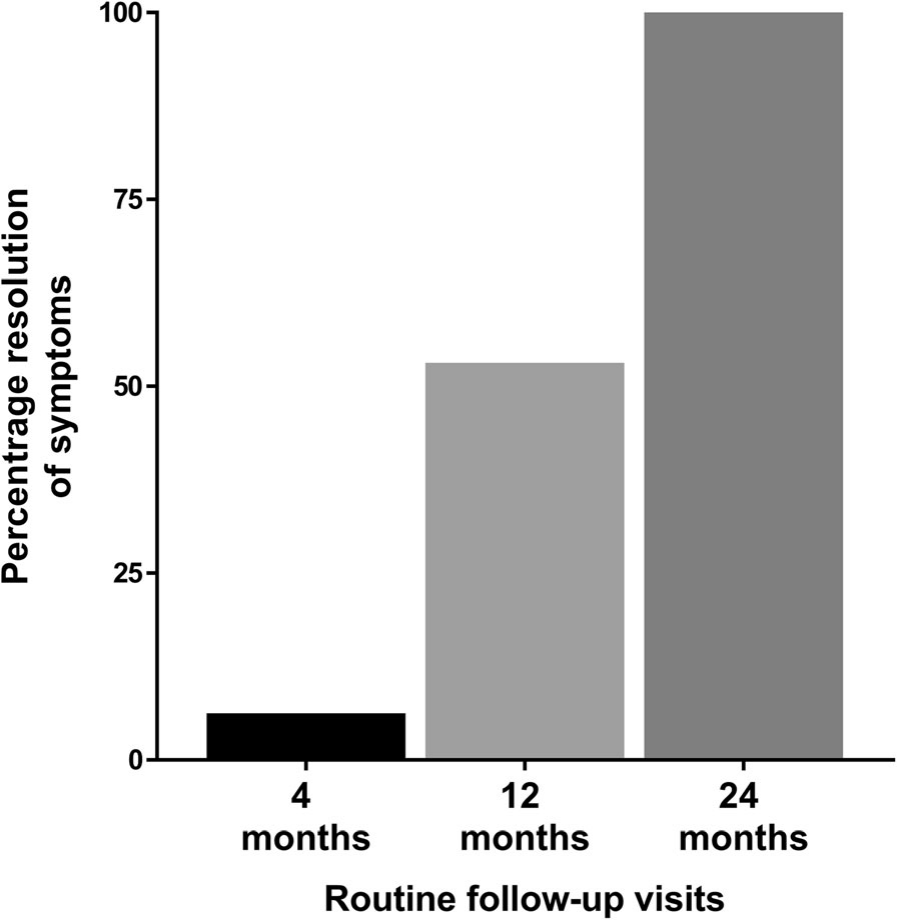
Percentage of patients reporting resolution of symptoms per follow-up visit presented for all patients (*N* = 32) that had a resolution of symptoms. Percentage of patients reporting resolution of symptoms per regular follow-up visit at 4, 12, and 24 months

In the total group of 152 non-converting patients, 38% (57 patients) indicated to have a resolution of symptoms after 2 years of follow-up and 63% (95 patients) had no symptom resolution. Similarly, in the group of 98 patients with serial imaging, 33% of patients (*n* = 32) reported resolution of symptoms whereas 67% (*n* = 66 patients) did not. In addition, in the 54 patients without serial MRIs, 25 experienced symptom resolution (46%) whereas 29 patients did not experience resolution of symptoms (54%). A chi-squared test comparing the number of patients experiencing symptom resolution in the groups of patients with and without serial MRI showed no significant difference (*p* = 0.09). The percentages of patients with complete clinical and imaging data indicating to experience resolution of symptoms (n = 32) at the follow-up visits at 4, 12, and 24 months are indicated in Fig. 2.

**Fig. 3:**
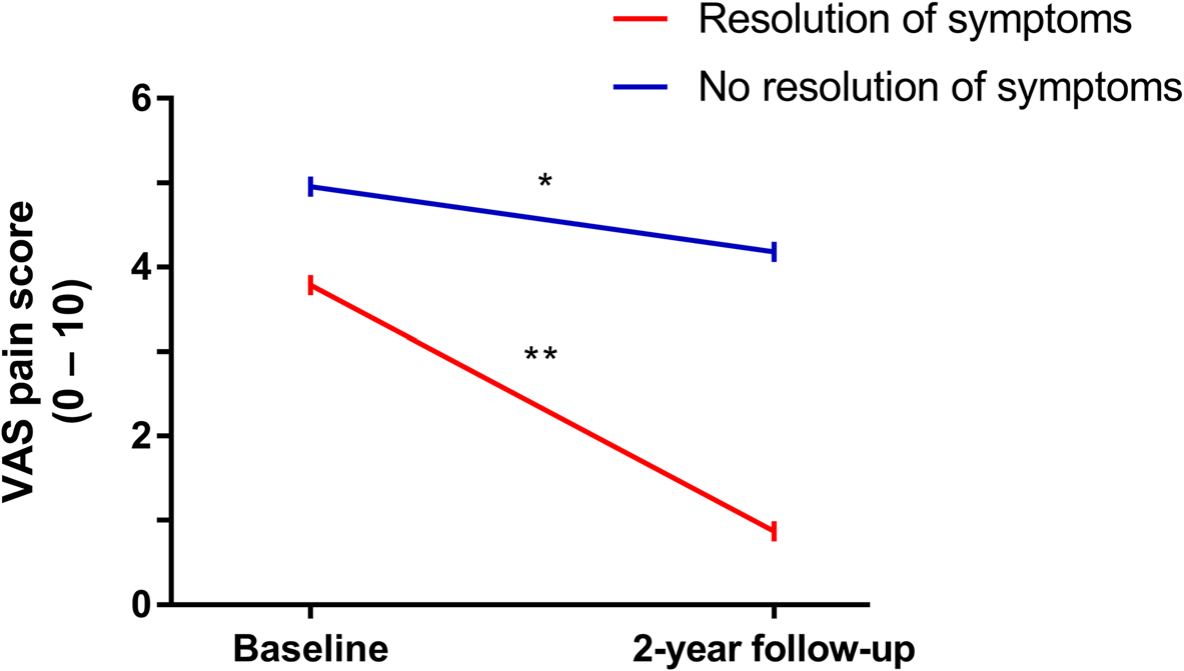
VAS pain scores over time for patients with and without resolution of symptoms (*N* = 98). *Significance at the *p* < 0.05 level, **significance at the *p* < 0.01 level

Within the patients that had complete clinical and MRI data, the patients that indicated to have a resolution of symptoms had a larger decrease in VAS pain scores over time than patients without a resolution of symptoms (decrease in VAS pain of 2.9 vs. 0.77; *p* < 0.001; Fig. 3). At baseline, the median 68-TJC was 4 in patients with a resolution and 6 in patients without resolution. After 2 years, the median TJC was 0 in patients with symptom resolution, whilst this was significantly higher in patients without symptom resolution (Mann-Whitney *U* test: *p* = 0.02). Several other characteristics of both groups evaluated at the 2-year follow-up are presented in Table 3. Although the resolution of symptoms was initially assessed with one question, these results show that patients that reported to have a symptom resolution improved in other measures for pain.

**Table 3:** Characteristics of the 98 patients with and without a resolution of symptoms at the 2-year follow-up

Patient characteristics	Symptom resolution (*n* = 32)	No symptom resolution (*n* = 66)	*p* value
68-TJC, median (IQR)	0 (0–0)	1 (0–4)	0.02
Presence of morning stiffness ≥ 60 min, *N* (%)	5 (16)	14 (21)	0.56
HAQ score, mean (SD)	0.18 (0.40)	0.60 (0.50)	0.09
VAS pain score, mean (SD)	0.87 (1.5)	4.2 (2.4)	< 0.001
VAS fatigue score, mean (SD)	3.7 (3.3)	5.6 (2.6)	0.003

*IQR* interquartile range, *SD* standard deviation, *TJC* tender joint count, *VAS* visual analogue scale (range 0–10)

Patients with remaining symptoms were diagnosed as persistent CSA because of persistent inflammatory type of arthralgia according to the rheumatologists (*n* = 43; 44% of all non-converters), osteoarthritis (*n* = 10; 10% of all non-converters), and tendinomuscular complaints (*n* = 13; 13% of all non-converters).

At disease presentation, the proportion of patients that used NSAIDs on a daily basis was equally distributed between patients with or without resolution of symptoms (22% vs. 23%; *p* = 0.89). After the 2-year follow-up, 9% of the patients with persistent symptoms used NSAIDs on a daily basis, whilst NSAIDs were not used in the group with symptom resolution, which is in line with the absence of symptoms.

### Clinical characteristics of patients with and without symptom resolution

Patients that later on achieved symptom resolution had no differences in baseline characteristics at baseline; Table 2 displays the patient characteristics for the 152 non-converting patients with complete clinical follow-up data, as well as the 98 non-converting patients with serial MRIs. The mean baseline total MRI inflammation score was slightly higher in patients that would eventually achieve symptom resolution (3.5) as compared to patients with persistent symptoms (2.7), but this was not statistically significant (*p* = 0.33).

### Association between symptom resolution and improvement of MRI inflammation

The mean total MRI inflammation scores of the 82 patients with a baseline total MRI inflammation score > 0 were compared to the MRI scores of similar age-matched symptom-free persons to infer if the MRI inflammation scores at the different time points exceeded the level of MRI-detected inflammation prevalent in the general population. Other characteristics of the symptom-free persons are provided in Additional file 1: Table S2.

**Fig. 4:**
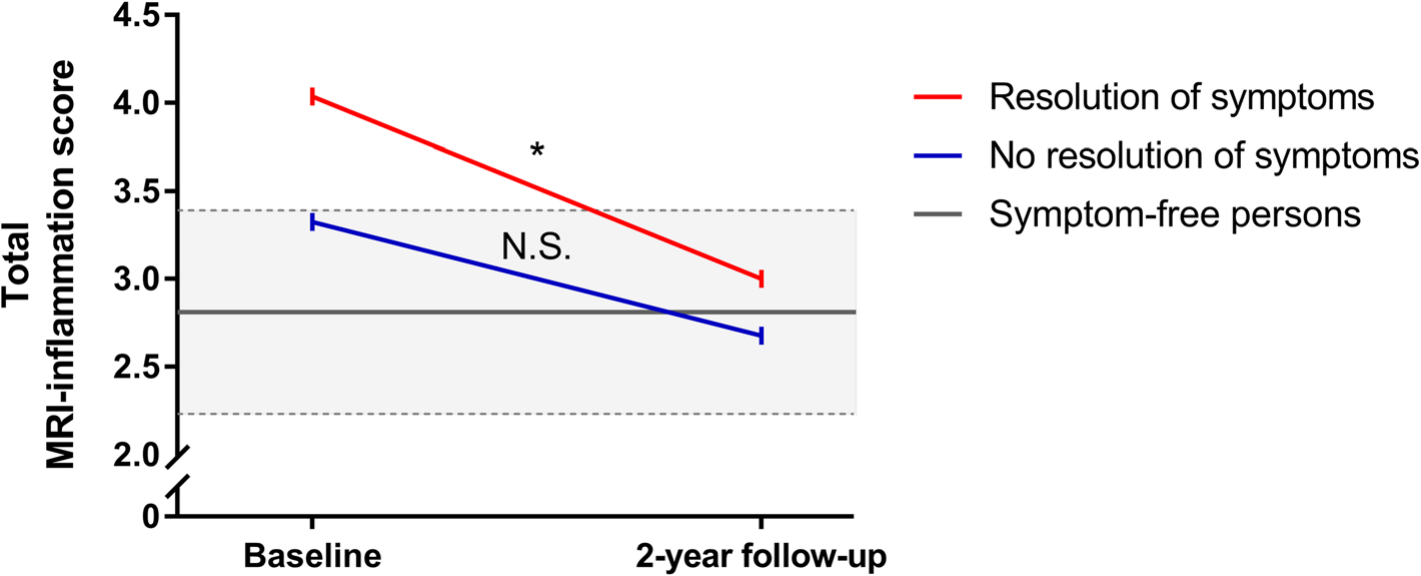
Mean total MRI inflammation scores over time for patients with and without resolution of symptoms (*N* = 82). The grey area indicates the mean and 95% confidence interval (dashed lines specify the upper and lower limit of the interval) of the total MRI inflammation score in age-matched symptom-free persons. Considered in this figure are patients with a baseline total MRI inflammation score > 0. At baseline, in the group without resolution of complaints, the mean total MRI inflammation score was not different as compared to symptoms-free persons (*p* = 0.26). Patients with resolution did have higher scores than symptoms-free persons (*p* = 0.04). After the 2-year follow-up, patients without and with a resolution of symptoms both did not have higher scores (*p* = 0.68 and *p* = 0.57, respectively). *Significance at the *p* < 0.01 level; N.S., non-significance

In the group of CSA patients that achieved resolution of symptoms over time, the mean MRI inflammation score was higher than that of symptom-free persons at baseline (4.0 vs. 2.6; *p* = 0.04; Fig. 4). In contrast, the patients that did not report a resolution of symptoms did not have higher MRI inflammation scores at baseline (mean 3.3 and 2.9; *p* = 0.26; Fig. 4).

After the 2-year follow-up, the mean total MRI inflammation score in patients with resolution of symptoms decreased to a level similar to that of symptom-free persons (3.0 vs. 2.6; *p* = 0.57; Fig. 4), whereas the patients without a resolution of symptoms still had no differences in their total MRI inflammation scores (mean 2.7 vs. 2.9; *p* = 0.68; Fig. 4). Comparison of the individual inflammatory features as detected by MRI are provided in Additional file 1: Figure S1; the decrease in the total MRI inflammation score was mostly due to a decrease in tenosynovitis and synovitis.

Finally, the difference of the total MRI inflammation scores over time was evaluated between baseline and 2-year follow-up (Fig. 4). The CSA patients with a resolution of symptoms had a statistically significant decrease in MRI inflammation score (difference 0.98; paired *t* test: *p* = 0.036). In the CSA patients that did not convert to RA and had no resolution of symptoms, the decrease was smaller (difference 0.44) and did not reach statistical significance (paired *t* test: *p* = 0.09).

Together, in patients with a resolution of symptoms, MRI inflammation scores were increased at the first presentation and normalized after symptom resolution, whereas patients that remained having symptoms (but did not progress to RA) did not have increased inflammation scores at any time point, with age-matched controls as a reference.

Although the group of patients without a resolution of symptoms was a heterogenous group in terms of final diagnosis, none of the separate diagnoses had a significant difference in MRI inflammation score over time: persistent CSA (*p* = 0.37), osteoarthritis (*p* = 0.60), and tendinomuscular complaints (*p* = 0.79). Separate matching of the patients with persistent CSA compared to symptom-free persons revealed no differences in the total MRI inflammation score at baseline (3.4 vs. 2.8; *p* = 0.25), or at the 2-year follow-up (2.6 vs. 2.8; *p* = 0.83). Matching of patients finally diagnosed with osteoarthritis and tendinomuscular complaints with symptom-free persons was not performed due to small patient numbers.

### Sub-analyses: autoantibody-positive patients

Although the presence of autoantibodies in CSA is associated with an increased risk of RA development, part of the patients with autoantibodies did not progress. In line with previous studies that reported a PPV of > 60% for ACPA-positive patients [1, 14] part of the autoantibody-positive patients did not progress to RA during the 2-year follow-up. In our data, 19% of the non-converting patients were either ACPA- or RF-positive. There was no conversion in ACPA or RF status in any direction over 2 years’ time.

Within the group of ACPA- or RF-positive non-converting patients (*n* = 19), 7 patients (37%) had symptom resolution over time and 12 patients (63%) had no resolution of symptom. The total MRI inflammation score decreased from 5.0 to 3.3 (difference 1.8; paired *t* test: *p* = 0.21) in patients with a resolution of symptoms. In patients without a resolution of complaints, the total MRI inflammation score reduced from 2.4 to 1.9 (difference 0.55; paired *t* test: *p* = 0.19). Comparison of MRI scores with symptom-free persons, as stratified by the resolution of symptoms, was not performed due to insufficient statistical power.

### Sensitivity analysis: patients meeting the EULAR definition

A sub-analysis was performed in patients that met the EULAR definition of arthralgia suspicious for progression to RA [13]. Sixty-four percent of the CSA patients that did not develop RA fulfilled the EULAR definition of arthralgia suspicious for progression to rheumatoid arthritis with ≥ 3 items present. Also in this subgroup, 37% of the patients achieved spontaneous resolution of symptoms.

Similar findings were obtained when patients meeting the EULAR definition and with a baseline total MRI inflammation score > 0 were compared to MRI scores of similar age-matched symptom-free persons. The patients experiencing resolution had higher MRI inflammation scores at disease presentation than symptom-free controls (*p* = 0.04), whilst the scores were no longer increased at the time of symptom resolution (*p* = 0.53). Patients without a resolution of symptoms (that did not progress to RA) did not have significantly increased MRI inflammation scores at any time point (Additional file 1: Table S3). Over time, MRI inflammation scores decreased in patients with symptom resolution (4.6 to 3.1; *p* = 0.02). In patients without a symptom resolution, scores did not decrease: 3.3 to 3.2; *p* = 0.67.

## Discussion

Patients with clinically suspect arthralgia are considered to be at risk for RA development by their rheumatologists. Most research done in the field of ‘RA risk’ is focussed on the subgroup of patients that indeed progress to RA. However, a large proportion of the patients that are considered to have an increased risk do not actually develop IA and RA. Here, we studied the group of non-converting patients and observed various outcomes. A considerable part of the patients that initially had presented with CSA continued to be characterized as CSA after a 2-year follow-up. A smaller part of the patients developed other explanations for their complaints. Interestingly, both latter groups of patients did not have increased MRI inflammation scores of small joints as compared to age-matched symptom-free persons. Furthermore, approximately one third of the non-converting patients had a resolution of symptoms over time. These patients had increased MRI-detected subclinical inflammation at baseline, which also resolved over time. This time relationship suggests that the subclinical inflammation was related to the presence of symptoms and the phenotype of CSA. In our view, this is the most interesting group of patients: these patients may indeed have been pre-RA but one of several final switches required for actual progression to RA were not turned ‘on’ and the disease process resolved without intervention.

Our study is the first to quantify the percentage of patients presenting with CSA that will have a resolution of symptoms over time. It consists of one third of all non-progressing patients and 27% of all patients that were identified as having CSA by rheumatologists. Interestingly, previous studies done in patients with UA showed that clinical synovitis resolved in 10–40% [3, 4], which is a similar range of spontaneous dissolvement. Similar as seen here, patients with spontaneous resolution were more often autoantibody-negative than patients with progression to arthritis. Despite the association with the absence of autoantibodies, the pathophysiologic mechanisms mediating spontaneous resolution or absence of progression are not elucidated yet. Our study served to identify this group of patients. Future studies are required to increase our understanding of the biological mechanisms involved.

This study had several limitations. First, patients were analysed during the 2-year follow-up, and patients that did not progress to RA could still develop IA after the follow-up of the study ended. However, as the Leiden University Medical Centre is the only referral centre in the region, it is unlikely that patients will visit another centre should symptoms reoccur. This allowed us to study if patients had returned to our Rheumatology Department after the formal final regular follow-up visit at 2 years. After an average of 5 years after the baseline visit, none of the patients had returned to be diagnosed with RA, indicating that patients truly did not develop RA. In addition, patients that had indicated that symptoms had disappeared after 2 years could theoretically experience renewed symptoms later on in life. However, this would not affect the current findings that resolution of symptoms was paralleled by resolution of subclinical inflammation.

A further limitation of our study is the small number of patients included. Especially the number of patients that were ACPA-positive and not progressed to RA is small, which warrants future studies with larger numbers of included patients to allow statistically more powerful analyses than our current, mostly exploratory, analyses.

Another limitation is that part of the patients did not complete the follow-up or did not consent to undergo another MRI. Although missing data was presumably not at random as patients with less severe symptoms are more likely to retract from the follow-up, the patient characteristics of the different groups were quite similar (Table 1), arguing against a major bias. However, the percentage of patients experiencing symptom resolution in the group that did not have complete imaging data over 2 years was slightly larger (46%) than the percentage of patients with complete imaging data (33%) which could be a potential source of bias, although the difference was not significantly different. Possibly, patients who experienced symptom resolution slightly less often felt the need to undergo imaging studies after 2 years.

Finally, since patients with baseline MRI scores of > 0 were studied, regression to the mean could have occurred. Furthermore, scores of MRI-detected inflammation were studied on a group level rather than joint level to decrease the possibility of type 1 error due to multiple testing. Nevertheless, we demonstrated that baseline scores in the patients with resolution significantly exceeded the level of MRI-detected subclinical inflammation of symptom-free persons, but not in the patients without resolution of symptoms.

The main outcome was a patient-reported resolution of symptoms. No validated questionnaire exists of patients with arthralgia at risk for RA, and we assessed this outcome using a single written question. The robustness of this outcome was illustrated by decreasing VAS pain scores and diminishing tender joint counts in the patients with a resolution, and therefore, we considered this to be a valid question that was interpreted well and uniformly by patients themselves.

Finally, DMARD therapy (including steroids) was not allowed and not prescribed during the course of the CSA study, but NSAIDs were allowed. NSAIDs were stopped before MR imaging. It could be questioned if NSAIDs played a role in disease resolution. However, NSAIDs are generally not considered as disease-modifying therapy, and the frequency of NSAIDs use at baseline was similar in patients with and without a symptom resolution.

In conclusion, one third of all patients with CSA that did not convert to IA or RA during the 2-year follow-up had resolution of symptoms and improvement of subclinical joint inflammation. This time relationship is suggestive for a causal relation of the subclinical inflammation and the phenotypic presentation of CSA. Further research is needed to identify the mechanisms that are involved in the resolution of disease processes.

## Supplementary information

**Additional file 1: Table S1.** Matrix of ICC scores of four readers who contributed to the data; each MRI was evaluated by two readers. **Table S2.** Characteristics of the matched symptom-free persons. **Table S3.** Comparison of mean MRI-inflammation scores of patients meeting the EULAR definition of arthralgia suspicious for progression to rheumatoid arthritis versus symptom-free persons. Methods. MRI scanning and scoring protocol. **Figure S1.** Mean scores of MRI-detected inflammatory features (Tenosynovitis, Synovitis, Bone Marrow Edema) over time for patients with and without resolution of symptoms.

## Data Availability

Data can be requested from the corresponding author.
