# Supplementary material for: Improvement of symptoms in clinically suspect arthralgia and resolution of subclinical joint inflammation: a longitudinal study in patients that did not progress to clinical arthritis
Source: Arthritis Res Ther. 2020 Jan 16;22:11. doi: 10.1186/s13075-020-2102-9 (PMC6966904; doi:10.1186/s13075-020-2102-9)
Supplement: Supplementary file 1 — Additional file 1: Table S1. Matrix of ICC scores of four readers who contributed to the data; each MRI was evaluated by two readers. Table S2. Characteristics of the matched symptom-free persons. Table S3. Comparison of mean MRI-inflammation scores of patients meeting the EULAR definition of arthralgia suspicious for progression to rheumatoid arthritis versus symptom-free persons. Methods. MRI scanning and scoring protocol. Figure S1. Mean scores of MRI-detected inflammatory features (Tenosynovitis, Synovitis, Bone Marrow Edema) over time for patients with and without resolution of symptoms. [file 13075_2020_2102_MOESM1_ESM.docx]

# List of Supplementary Files

*Supplementary Table 1. Matrix of ICC scores of four readers who contributed to the data; each MRI was evaluated by two readers*

*Supplementary Table 2. Characteristics of the matched symptom-free persons*

*Supplementary Table 3. Comparison of mean MRI-inflammation scores of patients meeting the EULAR definition of arthralgia suspicious for progression to rheumatoid arthritis versus symptom-free persons*

*Supplementary Methods. MRI scanning and scoring protocol*

*Supplementary Figure 1. Mean scores of MRI-detected inflammatory features (Tenosynovitis, Synovitis, Bone Marrow Edema) over time for patients with and without resolution of symptoms*

## Supplementary Table 1. Matrix of ICC scores of four readers who contributed to the data; each MRI was evaluated by two readers.

*Interreader intraclass correlation coefficients*

| **Reader** | **1** | **2** | **3** | **4** |
| --- | --- | --- | --- | --- |
| **1** | x | 0.97 | 0.97 | 0.97 |
| **2** | 0.97 | x | 0.99 | 0.95 |
| **3** | 0.97 | 0.99 | x | 0.94 |
| **4** | 0.97 | 0.95 | 0.94 | x |

*Intarreader intraclass correlation coefficients*

| **1** | **2** | **3** | **4** |
| --- | --- | --- | --- |
| 0.99 | 0.99 | 0.98 | 0.96 |

## Supplementary Table 2. Characteristics of the matched symptom-free persons

| Patient characteristic | Matched to patients without resolution of symptoms  (n=66) | | Matched to patients with resolution of symptoms  (n=32) | |
| --- | --- | --- | --- | --- |
|  |  |  |  |  |
| Age in years, mean (SD) | 47 | (13) | 46 | (14) |
| Female sex, N (%) | 53 | (80) | 20 | (63) |
| Symptom duration in weeks, median (IQR) | 0 | (0 – 0) | 0 | (0 – 0) |
| 68-TJC, median (IQR) | 0 | (0 – 0) | 0 | (0 – 0) |
| Increased CRP (≥5 mg/L), N (%) | n/a |  | n/a |  |
| Autoantibody status |  |  |  |  |
| Negative for IgM-RF and ACPA, N (%) | n/a |  | n/a |  |
| ACPA- or RF-positive, n (%) | n/a |  | n/a |  |

**Legend**:

ACPA = anti-citrullinated peptide antibody (positive if: ≥7 U/mL); CRP = C-reactive protein; IgM-RF = immunoglobulin M rheumatoid factor (positive if: ≥3.5 IU/mL); IQR = interquartile range; SD = standard deviation; TJC = tender joint count. N/A indicates not assessed as CRP, IgM-RF and ACPA were not tested in the symptom-free persons.

## Supplementary Table 3. Comparison of mean MRI-inflammation scores of patients meeting the EULAR definition of arthralgia suspicious for progression to rheumatoid arthritis versus symptom-free persons

|  | **Patients with resolution of symptoms** | **Patients without resolution of symptoms** | **Symptom-free persons** | **P-value** |
| --- | --- | --- | --- | --- |
|  | n=20 | n=34 | n=20 or n=34, respectively |  |
| **Baseline score** | 4.6 |  | 2.6 | 0.04 |
| **Score at two-year follow-up** | 3.1 |  | 2.6 | 0.53 |
|  |  |  |  |  |
| **Baseline score** |  | 3.3 | 3.2 | 0.45 |
| **Score at two-year follow-up** |  | 3.2 | 3.2 | 0.90 |

**Legend:**

Mean total MRI-inflammation scores for patients meeting the EULAR definition of arthralgia suspicious for progression to rheumatoid arthritis.[13] This table studied patients that fulfilled the EULAR definition and had baseline total MRI-inflammation score >0. Matching to symptom-free persons was performed in a 1:1 ratio. Please note that the symptom-free persons only had one MRI performed, and that the mean MRI-inflammation score was used as reference for both scans of the patients with CSA.

## Supplementary Methods. MRI scanning protocol and scoring

*Detailed MRI-scan protocol*

MR imaging was performed on an ONI MSK Extreme 1.5T extremity MR imaging system (GE Healthcare, Wisconsin, USA) using a 145mm coil for the foot and a 100mm coil for the hand. The patient was positioned in a chair beside the scanner, with the hand or foot fixed in the coil with cushions.

In the hand, the following sequences were acquired before contrast injection: T1-weighted fast spin-echo (FSE) sequence in the coronal plane (repetition time (TR) 575 ms, echo time (TE) 11.2 ms, acquisition matrix 388×288, echo train length (ETL) 2). After intravenous injection of gadolinium contrast (gadoteric acid, Guerbet, Paris, France, standard dose of 0.1 mmol/kg) the following sequences were obtained: T1-weighted FSE sequence with frequency selective fat saturation (fatsat) in the coronal plane (TR/TE 700/9.7ms, acquisition matrix 364×224, ETL 2), T1-weighted FSE sequence with frequency selective fat saturation in the axial plane (wrist: TR/TE 540/7.7 ms; acquisition matrix 320x192; ETL 2 and metacarpophalangeal joints: TR/TE 570/7.7 ms; acquisition matrix 320x192; ETL 2).

The obtained sequences of the forefoot were post-gadolinium sequences which included: T1-weighted FSE fatsat sequence in the axial plane (TR/TE 700/9.5ms; acquisition matrix 364x224, ETL 2) and: T1-weighted FSE fatsat sequence in the coronal plane (perpendicular to the axis of the metatarsals) (TR/TE 540/7.5ms; acquisition matrix 320x192, ETL 2). In the first 39 patients a T1-weighted sequence and a T2-weighted fat saturated sequence were acquired in the axial plane (relative to the anatomical position), before contrast agent administration at baseline. In the remaining 59 patients postcontrast, T1-weighted, fat saturated sequences were acquired in axial and coronal planes at baseline. This provided more information while reducing scanning-times. All patients underwent T1-weighted, fat saturated sequences in axial and coronal planes at two-year follow-up.

Field-of-view was 100mm for the hand and 140mm for the foot. Coronal sequences of the hand had 18 slices with a slice thickness of 2mm and a slice gap of 0.2mm. Coronal sequences of the foot had 20 slices with a slice thickness of 3mm and a slice gap of 0.3mm. All axial sequences had a slice thickness of 3mm and a slice gap of 0.3mm with 20 slices for the wrist, 16 for the metacarpophalangeal joints and 14 for the foot.

According to the RAMRIS-method, T2-weighted fat suppressed sequences, or when this sequence is not available a short tau inversion recovery (STIR) sequence, should be used to assess Bone Marrow (O)Edema (BME). Previously, three studies have demonstrated that a contrast enhanced T1-weigthed fat suppressed sequence has a strong correlation with T2-weighted fat suppressed sequences.[1–3] A T2-weighted image shows increased water signal and a contrast-enhanced T1-weighted sequence shows increased water content and the increased perfusion and interstitial leakage. A strong correlation has been shown in arthritis patients but also in patients without inflammatory diseases such as bone bruises, intraosseous ganglions, bone infarcts and even nonspecific cases.[2,3] We used the contrast enhanced T1-weighted fat suppressed sequence as it allowed a shorter scan time and has a higher signal to noise ratio. In the first 39 patients, baseline BME scores of the forefoot were evaluated using T2-weighted fat saturated sequence from the axial plane.

The tendons evaluated for tenosynovitis in the wrist were: extensor pollicis brevis, abductor pollicis longus; extensor carpi radialis brevis, extensor carpi radialis longus; extensor pollicis longus; extensor digitorum communis, extensor indicus proprius; extensor digiti quinti proprius; extensor carpi ulnaris; flexor carpi ulnaris tendon; flexor digitorum superficialis and profundus; flexor pollicis longus tendon and finally flexor carpi radialis. The tendons evaluated for tenosynovitis in the MCP joints were the flexor and extendor tendons of the MCP2 to MCP5. In the patients with available coronal planes of the forefoot, tenosynovitis was evaluated for flexor and extendor tendons of the MTP1 to MTP5.

*MR scoring*

All bones, joints and tendons were scored semi-quantitatively. Similar to the RAMRIS method, synovitis score was scored based on the volume of enhancing tissue in the synovial compartment (none, mild, moderate, severe (range 0-3)). Similar to method described by Haavardsholm et al the tenosynovitis-score was based on the thickness of peritendinous effusion or synovial proliferation with contrast enhancement (normal, <2mm, 2-5mm, >5mm (range 0-3)).[4,5] BME was depicted on a contrast enhanced T1-weigthed fat suppressed sequence and also scored on a 0-3 scale based on the affected volume of the bone (no BME, >0-33%, >33-66%, >66%). The scores of all joints were summed and the total BME, synovitis and tenosynovitis scored were summed as well, yielding the total MRI-inflammation-score.

**References**

1 Stomp W, Krabben A, Heijde D van der, *et al.* Aiming for a shorter rheumatoid arthritis MRI protocol: can contrast-enhanced MRI replace T2 for the detection of bone marrow oedema? *Eur Radiol* 2014;**24**:2614–22. doi:10.1007/s00330-014-3272-0

2 Schmid MR, Hodler J, Vienne P, *et al.* Bone Marrow Abnormalities of Foot and Ankle: STIR versus T1-weighted Contrast-enhanced Fat-suppressed Spin-Echo MR Imaging. *Radiology* 2002;**224**:463–9. doi:10.1148/radiol.2242011252

3 Mayerhoefer ME, Breitenseher MJ, Kramer J, *et al.* STIR vs. T1-weighted fat-suppressed gadolinium-enhanced MRI of bone marrow edema of the knee: Computer-assisted quantitative comparison and influence of injected contrast media volume and acquisition parameters. *J Magn Reson Imaging* 2005;**22**:788–93. doi:10.1002/jmri.20439

4 Østergaard M, Edmonds J, McQueen F, *et al.* An introduction to the EULAR–OMERACT rheumatoid arthritis MRI reference image atlas. *Ann Rheum Dis* 2005;**64**:i3–7. doi:10.1136/ard.2004.031773

5 Haavardsholm EA, Østergaard M, Ejbjerg BJ, *et al.* Introduction of a novel magnetic resonance imaging tenosynovitis score for rheumatoid arthritis: reliability in a multireader longitudinal study. *Ann Rheum Dis* 2007;**66**:1216–20. doi:10.1136/ard.2006.068361

## Supplementary Figure 1. Mean scores of MRI-detected inflammatory features (Tenosynovitis, Synovitis, Bone Marrow Edema) over time for patients with and without resolution of symptoms


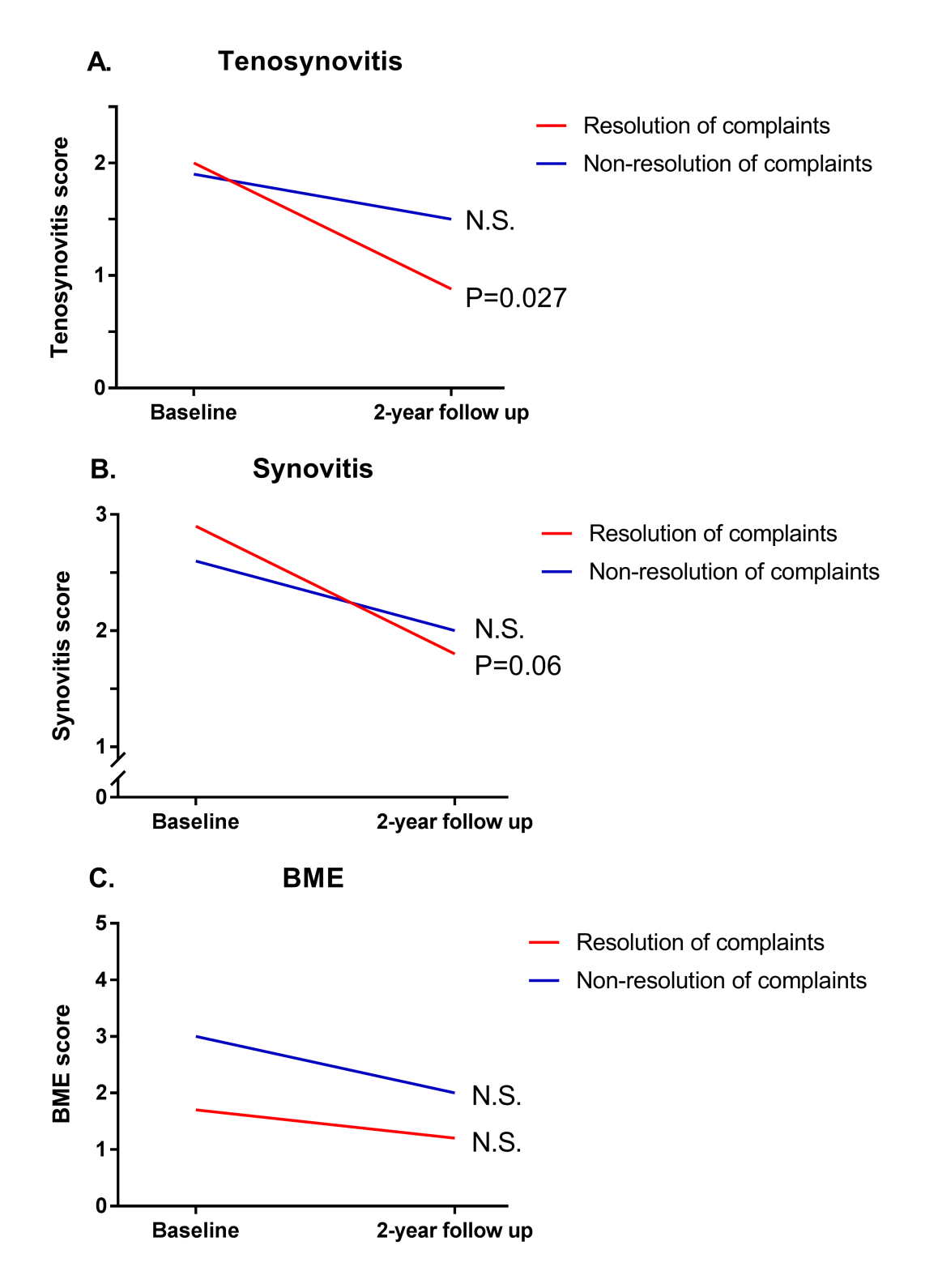


**Legend**:

BME = Bone Marrow Edema; N.S. indicates non-significance at the P < 0.05 level.
